# Epithelial to Stromal Re-Distribution of Primary Cilia during Pancreatic Carcinogenesis

**DOI:** 10.1371/journal.pone.0164231

**Published:** 2016-10-26

**Authors:** Simon Schimmack, Sarah Kneller, Nigora Dadabaeva, Frank Bergmann, Andrew Taylor, Thilo Hackert, Jens Werner, Oliver Strobel

**Affiliations:** 1 Department of General, Visceral and Transplantation Surgery, Heidelberg University Hospital, Im Neuenheimer Feld 110, 69120 Heidelberg, Germany; 2 Institute of Pathology Heidelberg, Im Neuenheimer Feld 224, 69120 Heidelberg, Germany; 3 University Hospital of General, Visceral, Transplantation, Vascular and Thoracic Surgery of Munich, Campus Großhadern, Marchioninistraße 15, 81377 Munich, Germany; Vrije Universiteit Brussel, BELGIUM

## Abstract

**Background:**

The Hedgehog (HH) pathway is a mediator in pancreatic ductal adenocarcinoma (PDAC). Surprisingly, previous studies suggested that primary cilia (PC), the essential organelles for HH signal transduction, were lost in PDAC. The aim of this study was to determine the presence of PC in human normal pancreas, chronic pancreatitis, and during carcinogenesis to PDAC with focus on both epithelia and stroma.

**Methods:**

PC were analyzed in paraffin sections from normal pancreas, chronic pancreatitis, intraductal papillary-mucinous neoplasia, and PDAC, as well as in primary human pancreatic stellate cells (PSC) and pancreatic cancer cell lines by double immunofluorescence staining for acetylated α-tubuline and γ-tubuline. Co-staining for the HH receptors PTCH1, PTCH2 and SMO was also performed.

**Results:**

PC are gradually lost during pancreatic carcinogenesis in the epithelium: the fraction of cells with PC gradually and significantly decreased from 32% in ducts of normal pancreas, to 21% in ducts of chronic pancreatitis, to 18% in PanIN1a, 6% in PanIN2, 3% in PanIN3 and to 1.2% in invasive PDAC. However, this loss of PC in the neoplastic epithelium is accompanied by a gain of PC in the surrounding stroma. The fraction of stromal cells with PC significantly increased from 13% around normal ducts to about 30% around PanIN and PDAC. HH-receptors were detected in tumor stroma but not in epithelial cells. PC are also present in PSC and pancreatic cancer cell lines.

**Conclusion:**

PC are not lost during pancreatic carcinogenesis but re-distributed from the epithelium to the stroma. This redistribution may explain the re-direction of HH signaling towards the stroma during pancreatic carcinogenesis.

## Introduction

Pancreatic ductal adenocarcinoma (PDAC) is the fourth most frequent cause of death from malignant diseases in the US [1]. The majority of tumors are in the advanced stages at diagnosis [2] since symptoms are unspecific, which impedes early detection [3]. Invasive PDACs occur from the precursor lesions in pancreatic intra-epithelial neoplasia (PanIN) or intraductal papillary-mucinous neoplasia (IPMN) [4], similar to the adenoma-carcinoma sequence of colorectal cancer [5]. Pathognomonic for this disease is a stromal reaction, desmoplasia, which occurs during tumor progression, extensively employing fibroblasts, pancreatic stellate cells (PSC) and the extracellular matrix [6]. The cells of origin are unknown but according to recent literature, acinar cells [7, 8] and ductal epithelial cells [8, 9] are potential sources.

These cells, like most vertebrate cell types (http://www.bowserlab.org/primarycilia/cilialist.html) [10], exhibit cilia [11, 12], versatile excrescences of the cell membrane [13] that exist in motile and immotile forms. Motile cilia generate luminal transport by their movements and occur in vast numbers on the cell surface; the latter, immotile variety are called primary cilia (PC) and each cell typically has only a single exemplar [14]. PC are now considered to be the central organelles for intercellular communication [13] and have been reported to play a critical role in many diseases [15] and to be lost in many different cancer types [16–22], including PDAC [23].

**Fig 1:**
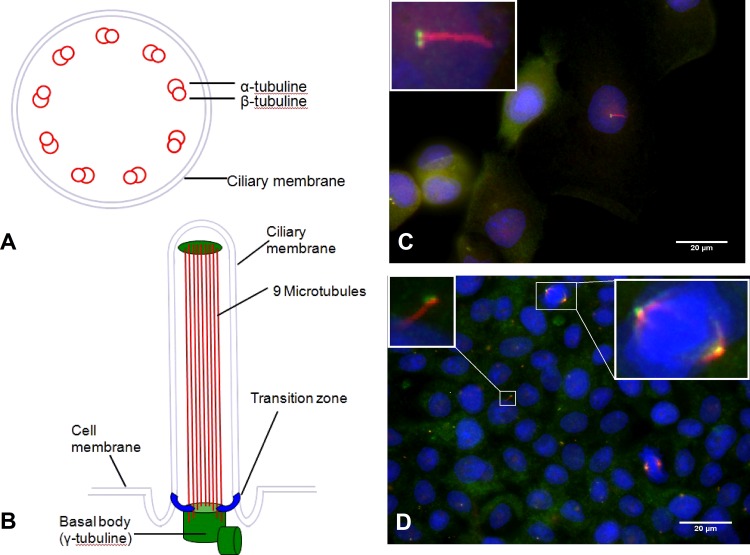
Schematical structure and visualization of primary cilia (PC). **(A)** Cross section of primary cilia, consisting of 9 microtubule duplets containing α and β tubline. **(B)** Structure of PC. Cilia are fixed by basal bodies on the cell membrane. **(C)** Immunofluorescent visualization of PC in Panc1 cells. The axonem is stained in red (acetylated α-tubline), the basal body is stained green (γ-tubline). **(D)** Immunofluorescent visualization of PC and the spindle apparatus in the BxPx3 cancer cell line.

The structure of PC is shown in **Fig 1**. The basal body (two centrioles) contains nine microtubuli (MT)-triplets as well as γ-tubuline and is considered to anchor the long axonem [24]. The axonem is the matrix of the PC and is represented by nine MT-duplets, consisting of α- and β-tubuline-monomers [24] connected through nexin [13, 25]. Consequently, γ-tubuline for the basal body [26] and acetylated α-tubuline for the axonem [27] have been used as target proteins in cilia for the detection by immunohistochemistry (**Fig 1A**). During the cell cycle, PC are absorbed [28] as the two centrioles move to the cell pole and organize mitosis as part of the spindle apparatus [13, 24] (**Fig 1C**).

As organelles for signal transduction, PC are a unique site of expression of receptors for several pathways [14, 25], including the Hedgehog (HH)-receptor Patched1 (PTCH1) [29]. Subsequently, if the PC is disrupted, HH signaling has shown to be impossible [30, 31]. Furthermore, it has been demonstrated that HH signaling plays a critical role in pancreatic neoplasms [32], since HH-receptor ligands are over-expressed in PDAC [32, 33] as well as in malignant IPMN [34]. This over-expression promotes increased proliferation and reduced apoptosis in pancreatic cancer cells lines [33]. Miss-expression of HH ligands leads to metaplasia and therefore, may play a role in PDAC carcinogenesis [9].

Seeley and colleagues have reported that PC are lost in both PanIN and PDAC [23], which runs seemingly counter to the evidence of over-activation of the HH signaling pathway in PDAC [33], since HH-receptors are localized on PC. This study has attempted to resolve this inconsistency by characterizing the presence of primary cilia and HH signaling pathway proteins in the epithelium and stroma during the progression of human pancreatic cancer.

## Material and Methods

### Human sample collection

Paraffin embedded pancreatic tissues from healthy pancreas (n = 6), patients with chronic pancreatitis (n = 7), benign IPMN (n = 4), malignant IPMN (n = 8), patients with G1/G2 pancreatic ductal adenocarcinomas (PDAC, n = 13) and G3/G4 PDACs (n = 12) containing PanIN lesions were obtained from the Biobank of the European Pancreas Center in Heidelberg after collection according to the Ethics Committee requirements for the University of Heidelberg, Germany (ethic vote No. 301/2001, amendment 2014). All patients provided their written consent. Healthy pancreatic tissue was obtained from pancreata of multiple-organ donors which were not used for transplantation. “Benign” IPMN were defined as IPMN with low or moderate dysplasia, while “malignant” IPMN showed features of carcinoma *in situ* or invasive carcinoma. In order to re-validate the pathological diagnosis, the middle section (thickness 4 μm) out of the 11 sections exclusively assembled for this project (using Microtom, RM2255, Leica, Bensheim, Germany) was stained after de-paraffinization with hemotoxylin (Merck Bioscience, Schwalbach, Germany) and eosin (Merck Bioscience, Schwalbach, Germany) for examination by an experienced pancreatic pathologist (FB).

### Cell lines

The human pancreatic adenocarcinoma cell lines BxPc3, Capan1, MiaPaCa2, and Panc1 were obtained from the American Tissue Type Culture Collection (ATCC, Rockville, MD, USA). All cell lines were verified by the German Collection of Micro-organisms and Cell cultures (DSMZ) at the Leibnitz-Institute using gene-profiling and cultivated in medium containing 90% RPMI 1640 (Gibco, Grand Island, NY) and 10% FBS (Pan Biotech, Aidenbach, Germany) in 75 cm^2^ flasks (Sarstedt, Newton, NC) at 37°C and 5% CO_2_.

### Primary human pancreatic stellate cells

Primary human pancreatic stellate cells (PSC) were isolated from intra-operatively obtained human pancreatic ductal adenocarcinoma tissue and cultured as previously described [25].

### Double immunofluorescence staining

Double immunofluorescence staining was performed following previously described protocols [8]. For detection of primary cilia, a 1:3000 dilution of mouse anti-acetylated α-tubuline (T6793, DAKO, Hamburg, Germany) and a 1:500 dilution (1:250 dilution for PSC) of mouse anti-γ-tubuline (T6557, DAKO, Hamburg, Germany) primary antibodies were used. The primary anti-γ-tubuline antibody was linked to the secondary antibody Alexa flour 488 (1:333, Invitrogen, Carlsbad, CA), which visualized γ-tubuline as green dots within the centriols at the base of PC. The primary anti-acetylated α-tubuline antibody was linked to the secondary antibody Alexa flour 568 (1:333, Invitrogen, Carlsbad, CA) resulting in red fluorescent staining of the entire axionem of PC. The primary antibody FLEX (DAKO, Hamburg, Germany) was used as a negative control for unspecific binding. The secondary antibody was applied together with 4’,6-diamidino-2-phenylindole (DAPI, Sigma-Aldrich, Saint-Louis, MO, USA) and 0.25% Triton X (Merck Bioscience, Schwalbach, Germany) to visualize the nuclei. Immunofluorescent staining was assessed with an Axioplan 2 Imaging Microscope (Carl Zeiss AG, Oberkochen, Germany) using the Axiovision Rel. 4.8 software. This software provides 4 single images from planes differing by 1 μm thickness which are combined three-dimensionally, allowing for digital evaluation of tissue thickness up to 3 μm, called “Z-decks”. Pictures were taken by the Axiocam HRc camera (Carl Zeiss AG, Oberkochen, Germany). The length and fraction of PC were evaluated with ImageJ (NIH, USA).

### Cells and PC counting

For every piece of tissue, at least three high-power-fields of interest (Z-decks) were taken and analyzed using a magnification of 63 x. In donor tissue and chronic pancreatitis, all visible duct epithelial cells and their primary cilia of at least three intra- and interlobular ducts (median counted cells/Z-deck: 26.5, range: 14–63), as well as the corresponding stromal area (median counted cells/Z-deck: 30, range: 10–62) were counted and analyzed. In PDAC tissue, 3–6 high-power-fields of interest were taken and analyzed (37.6 stromal cells/Z-deck, range: 11–68; 61.9 tumor cells/Z-deck, range: 19–110). Depending on the frequency of PanIN lesions within a tumor or CP, between one and eight PanIN lesions were analyzed per tissue.

### Statistical Evaluation

All statistical analyses were performed using Microsoft Excel and Prism 5 (GraphPad Software, San Diego, CA). Binary comparisons were made using a 2-tailed Mann-Whitney Test. Comparisons between more than 2 groups were performed using the Kruskal Wallis test, followed by the Dunn post-hoc test where appropriate. A *p*-value of < 0.05 was designated as significant. Statistical significance is indicated by an asterisk or rhomb and described in the figure legends.

## Results

### Length of epithelial and stromal primary cilia and fraction of cilia carrying cells in different pancreatic tissues

**Fig 2:**
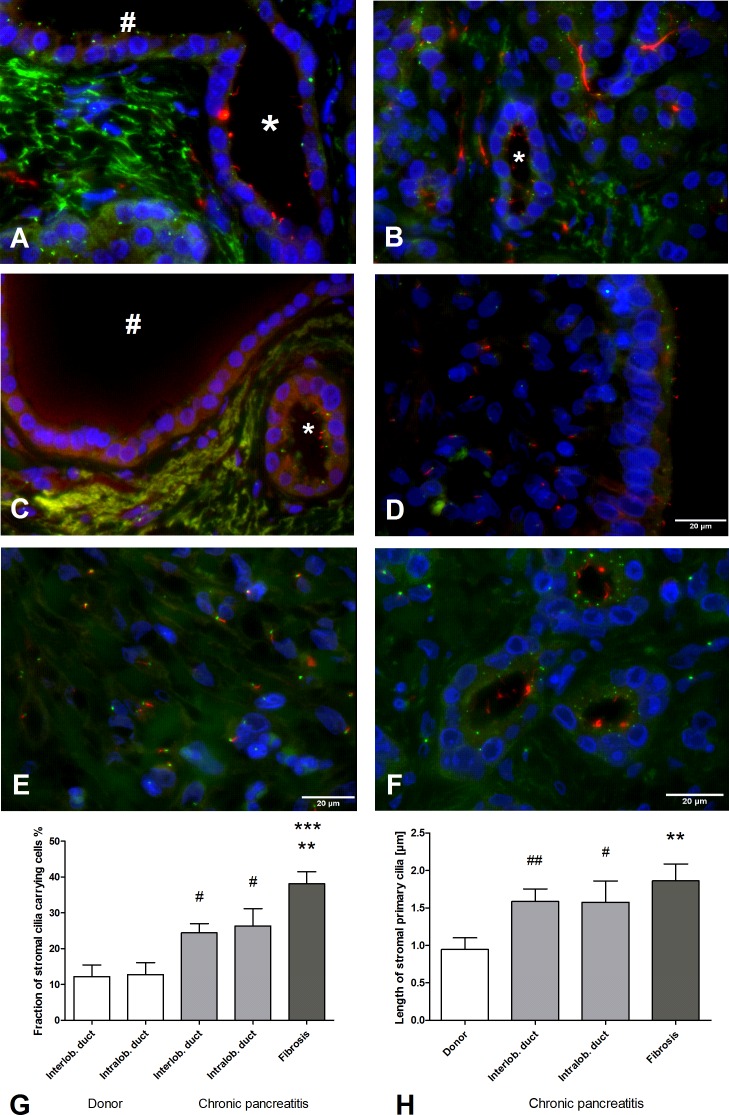
Primary cilia (PC) in healthy pancreas and chronic pancreatitis (CP). **(A)** Intralobular duct (*) with epithelial PC. Towards larger ducts (#), only basal bodies are stained, the axonem is not developed. **(B)** PC-rich acinar region around an intralobular duct (*). **(C)** No significant expression of PC in the interlobular duct (#) while epithelial cells in smaller intralobular ducts (*) show PC. **(D)** Little development of PC in pancreatitis ducts. In CP, an increase of stromal PC length and number is observed in comparison to healthy pancreatic stroma around ducts (see also **(G)** and **(H)**). **(E)** In fibrotic areas of CP tissue, length and number of stromal PC is further increased (see also **(G)** and **(H)**. **(F)** Tubular complexes also contain many PC. **(G)** Fraction of stromal PC carrying cells is higher in CP tissue than in normal pancreatic tissue (donor) in all areas assessed (Kruskal-Wallis test: *p* < 0.001, post-hoc Dunns test: ***p<0.001 vs intralobular duct (donor), **p<0.01 vs interlobular duct (donor), Mann-Whitney test: #*p* < 0.05 vs. donor ducts). **(H)** Stromal PC are significantly longer in the stroma of CP compared to normal pancreas (Kruskal-Wallis test *p* < 0.01, post-hoc Dunns test **p < 0.01 vs. donor, Mann-Whitney test: ##*p* < 0.01, #*p* < 0.05 vs. donor). Acetylated α-tubuline: red. γ-tubuline: green. DAPI: blue. Mean ± SEM.

In a normal pancreas, epithelial PC were present in 31.8% of intralobular and terminal pancreatic ducts (**Fig 2A**), and exhibited in 15.5% in interlobular ducts. This distribution was accompanied by a shortening of epithelial PC towards bigger ducts (intralobular ducts: 1.5 μm, interlobular ducts: 1.0 μm) (*data not shown*). Stromal cells were rarely ciliated (12%) and the stromal cell PC were short (0.9 μm, **Fig 2**).

Epithelial PC were present in only 20.6% of intralobular ducts in chronic pancreatitis (CP), a significant decrease in comparison to normal pancreas (31.8%, *p* < 0.05, *data not shown*). In addition, the length of the PC decreased from intralobular to interlobular ducts (1.6 μm and 1.0 μm, respectively, *data not shown*). There was a comparative increase from 12 to 25% of ciliated cells around the ducts in CP stromal tissue to normal stroma (*p* < 0.05, **Fig 2B and 2E**). In fibrotic areas, up to 38.1% of all stromal cells were ciliated (*p* < 0.01, **Fig 2C and 2E**). The length of PC in CP stromal cells (1.9 μm) was increased in comparison to normal stroma (0.9 μm, p < 0.01, **Fig 2F**).

**Fig 3:**
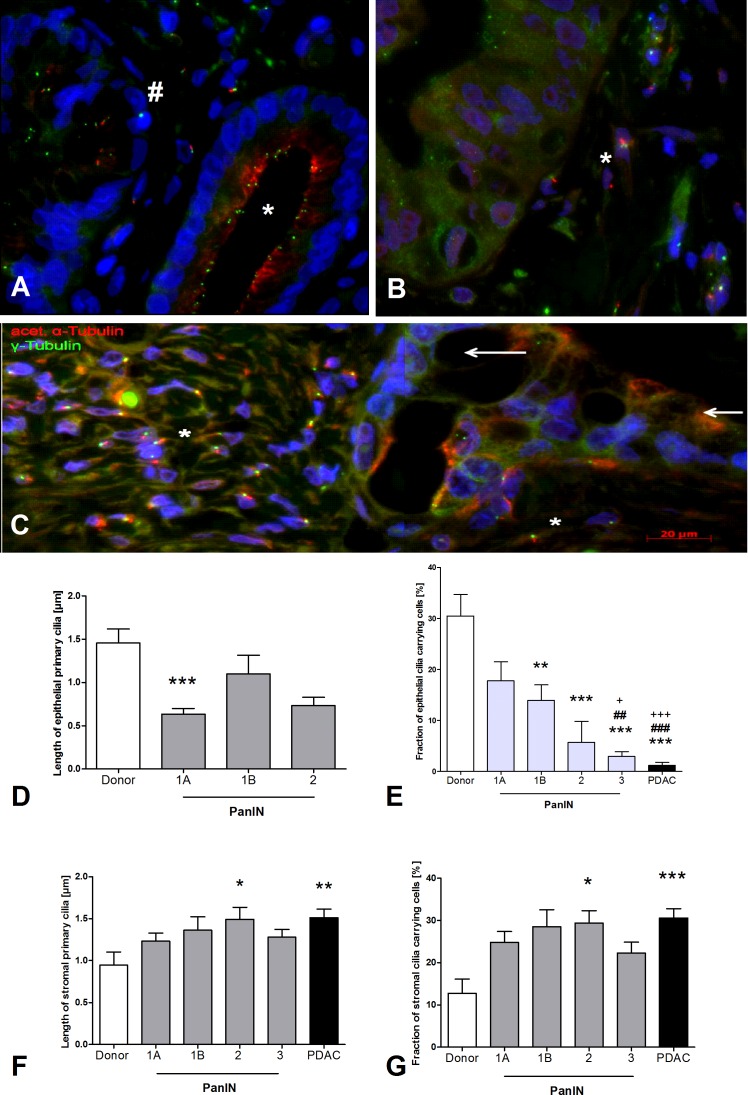
Primary cilia (PC) in pancreatic intra-epithelial neoplasia (PanIN) and pancreatic cancer cells (PDAC). **(A)** Comparison between PanIN 1a (*) and PanIN 1B (#), the latter showing papillary epithelium and reduced number of cilia. **(B)** PanIN 3 lesion. Epithelial cells do not carry cilia while PC are present in stromal cells (*). **(C)** Loss of epithelial PC in PDAC (indicated by arrows), while in the stromal cells there is a noticeable increase of both the length of PC and the number of PC carrying cells (*). **(D)** Length of epithelial PC is decreased in PanIN lesions in comparison to normal pancreas (donor). **(E)** Gradual loss of epithelial PC in PanIN lesions. In PDAC, almost no epithelial PC were detected. **(F)** In stromal tissue around PanIN lesions and PDAC (G1/G2), an increased length of PC and **(G)** increased fraction of cilia carrying cells was evident compared to normal pancreas (donor). Kruskal-Wallis test: *p* < 0.0001, post-hoc Dunns test: ***p < 0.001 vs. donor, **p < 0.01 vs donor, *p < 0.01 vs donor, ###*p* < 0. 001 vs PanIN 1A, ##*p* < 0. 01 vs PanIN 1A, +++*p* < 0. 001 vs PanIN 1B, +*p* < 0. 05 vs PanIN 1B, acetylated α-tubuline: red, γ-tubuline: green, DAPI: blue. Mean ± SEM.

In comparison to healthy pancreas, fewer and shorter PC developed in neoplastic pancreatic tissue (**Fig 3**). During PanIN progression, there was a gradual loss of epithelial PC from 17.8% PanIN 1A, to 14% PanIN 1B, 5.7% PanIN 2, to 2.9% PanIN 3 (*p* < 0.001, **Fig 3A, 3B and 3F**). However, this loss of epithelial PC was accompanied by a corresponding increase of PC in the stroma. This fraction of ciliated cells surrounding PanIN increases from 24.8% PanIN 1A, to 28.5% PanIN 1B, to 29.4% PanIN 2, to 22.3% PanIN 3 (*p* < 0.001, **Fig 3H**). The length of stromal PC around PanIN also increased significantly in comparison to the length of PC in normal stroma (1.5 μm vs. 0.9 μm, *p* < 0.05, **Fig 3G**). In contrast, the length of epithelial PC in neoplastic cells, such as PanIN 1A, decreased comparatively to PC in normal ductal cells (*p* < 0.001, **Fig 3E**).

In 13 invasive G1/G2 pancreatic ductal adenocarcinomas (PDACs), only 1.2% of epithelial cells were ciliated while 31.5% of stromal cells had PC (**Fig 3C, 3D, 3F and 3H**). 11 PDAC expressed no epithelial PC (n = 7) or very few epithelial PC (n = 4, 0.96–2.3%). Two PDAC demonstrated a higher expression of PC (4.65 and 5.85%) which was still markedly lower than in healthy pancreas, CP and PanIN1 (see Fig 3E). Despite performing double immunofluorescent staining in 12 G3/G4 PDAC, an adequate analysis of PC was not possible due to the high fraction of undifferentiated cells precluding the distinction between epithelial and stromal cells.

**Fig 4:**
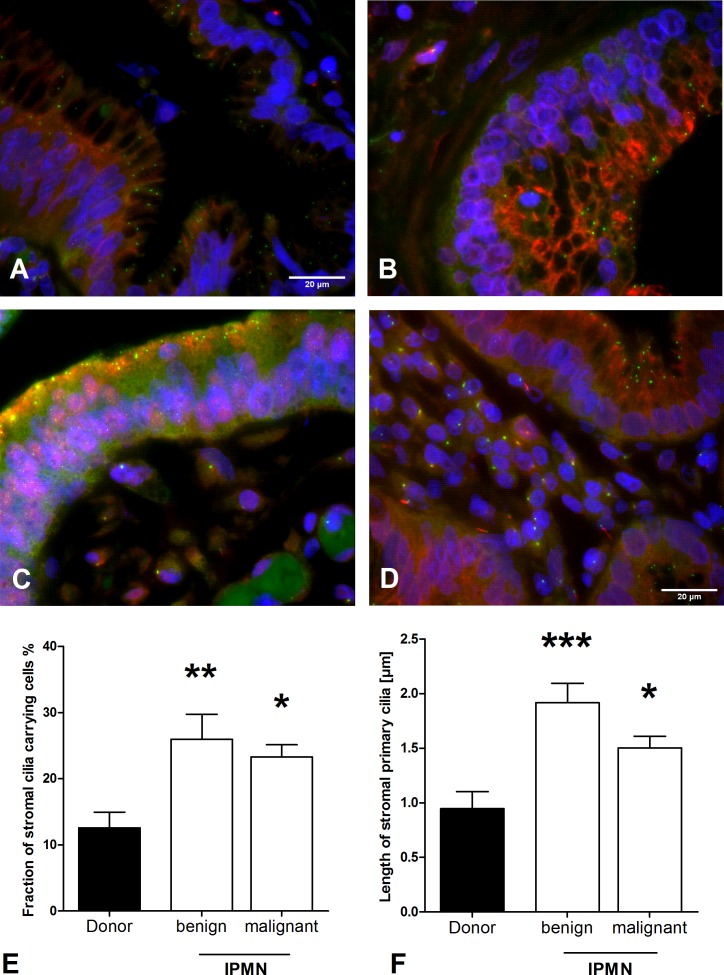
Primary cilia (PC) in intraductal papillary-mucinous neoplasia (IPMN). **A/B)** Two benign and **(C)** one malignant IPMN without PC. **(D)** In stromal tissue surrounding IPMN lesions, an increased fraction of cilia carrying cells (**E**, Kruskal-Wallis test: *p* < 0. 01) and an increased length of PC (**F**, Kruskal-Wallis test: *p* < 0. 001) were detected compared to normal pancreas (donor). Post-hoc Dunns test: *p < 0.05, **p < 0.01, ***p < 0. 001, acetylated α-tubuline: red, γ-tubuline: green, DAPI: blue. Mean ± SEM.

Four IPMN as PDAC precursor lesions, as well as 8 malignant IPMN were also examined and no PC were detected in benign (**Fig 4A and 4B**) and malignant (**Fig 4C**) IPMN. Similar to other examined pancreatic lesions, ciliated cells in IPMN-surrounding-stroma show a significant increase (*p* < 0.01, **Fig 4C and 4E**) with one (8.3%) exception. Consistent with benign IPMN, no PC were found in malignant IPMN (**Fig 4D**). Additionally, the length of PC was elevated in the stroma of malignant (1.5 μm) and benign IPMN (1.9 μm) in comparison to the normal inter-lobular ducts surrounding the stroma (0.9 μm, *p* < 0.001, **Fig 4F**).

### Re-distribution of PC from epithelial to stromal cells in pancreatic carcinogenesis

**Fig 5:**
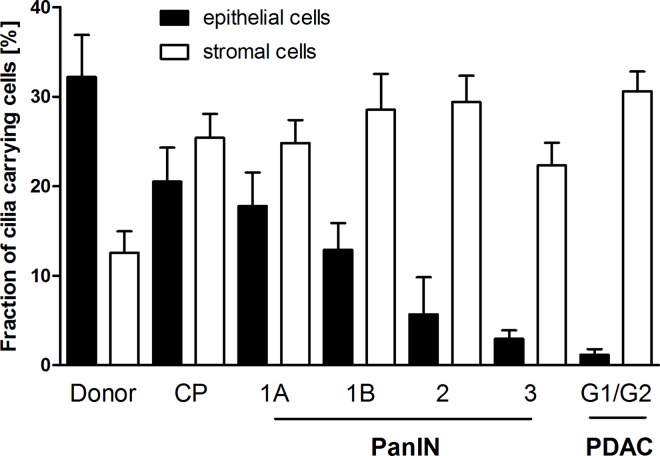
Re-Distribution of primary cilia (PC) from epithelial to stromal cells in pancreatic carcinogenesis. The fraction of primary cilia (PC) carrying cells decreases in epithelia, while there is a simultaneous increase of ciliated cells in stromal tissue during pancreatic carcinogenesis (from PanIN 1A to pancreatic G1/G2 ductal adenocarcinomas (PDAC). Mean ± SEM.

The data demonstrates not only a gradual loss of epithelial primary cilia but an associated gain of primary cilia in stromal cells, resulting in an overall re-distribution of PC from the epithelium to the stroma during pancreatic carcinogenesis (**Fig 5**). This suggests a role of PC in desmoplasia for pancreatic ductal adenocarcinoma.

### Localization of the Hedgehog receptor components patched (PTCH) and smoothened (SMO)

**Fig 6:**
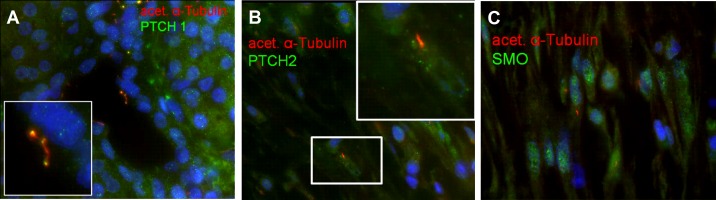
Localization of Hedgehog-signaling pathway receptors PTCH1, PTCH2 and SMO. **(A)** PTCH1 was localized on PC of normal epithelial cells while **(B)** PTCH2 and **(C)** SMO were mostly present in stromal PC of pancreatic ductal adenocarcinoma.

Given the PC re-distribution from epithelium to the stroma, the PC-linked Hedgehog (HH) signaling in epithelial and stromal cells was then examined. Double-staining with acetylated α-tubulin and the HH receptor proteins PTCH1, PTCH2 and SMO, which are essential for HH-signal transduction was performed. In normal pancreas, PTCH1 was expressed on epithelial cells (**Fig 6A**) with decreasing density towards interlobular ducts (*data not shown*), mirroring the distribution observed for PC (see **Fig 2G**), whereas in neoplastic epithelia, PTCH1 was lost (*data not shown*).

In contrast, in the normal pancreas the PTCH2 receptor was not expressed in epithelial cells but in stromal cells, with the strongest expression in the stroma surrounding the neoplastic epithelium (**Fig 6B**). SMO was only detected in the stromal cells of PDACs (**Fig 6C**).

### Primary cilia on pancreatic stellate cells and cancer cell lines *in vitro*

**Fig 7:**
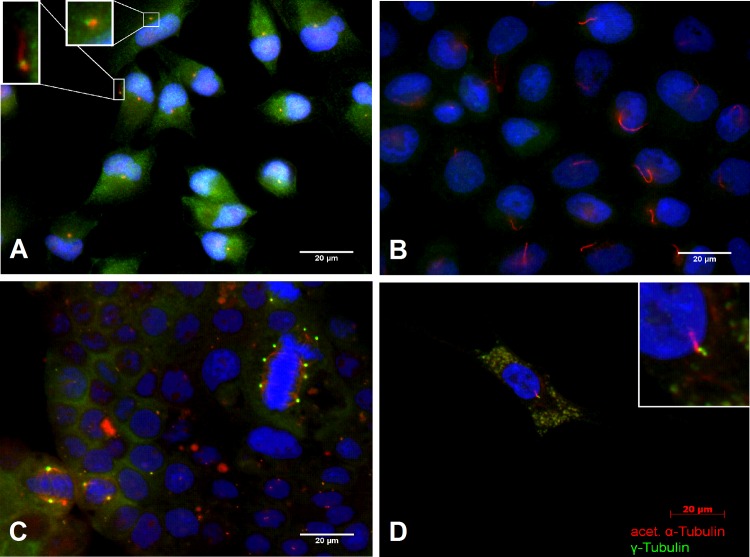
PC in pancreatic stellate cells (PSC) and pancreatic cancer cell lines. **(A)** MiaPaCa2 cells developing sporadic PC. **(B)** In these starving Panc1 cells, PC are expressed more frequently. **(C)** Multiple mitosis can be seen in Capan1 cells. The spindle apparatus never appears together with PC, inferring it is reabsorbed during mitosis. **(D)** PC are frequently present even in isolated PSC.

Given that immunohistochemical images are momentary snapshots in tissue, the human pancreatic cancer cell lines BxPc3, Capan1, MiaPaCa2, and Panc1 were used for a more dynamic representation of PC expression, demonstrating the sporadic formation of PC (**Fig 7A**). Since the culture media used was not typical of the tumor microenvironment, a more authentic, and thus, competitive tumor environment was simulated with low oxygen and nutrition by seeding Panc1 cells in a high concentration and incubating them for 7 days without changing the media. Under these competitive conditions, cells showed enhanced formation of PC as demonstrated in **Fig 7B,** confirming that PC are not lost in pancreatic cancer cells lines [35]. Furthermore, it indicates that PC may even play a role in adapting to lack of nutrition or oxygen. Staining of PC and spindle apparatus was never detected in the same cell confirming that PC are absorbed during mitosis (**Fig 7C**).

Given the PC re-distribution from the epithelium to the stroma, and since pancreatic stellate cells (PSC) play an important role with regard to the desmoplastic stroma-reaction in PDAC [36], we next examined whether PSC express PC and therefore may be able to transfer HH signals. Immunohistochemistry demonstrated that PC are formed by PSC as shown in **Fig 7D.**

## Discussion

Pancreatic cancer is a malignancy in which over-activation of HH signaling plays an important role [23]; hence it is surprising that previous studies have indicated a loss of primary cilia (PC), the central organelles for HH pathway transduction in the disease [33]. In this study, we have demonstrated a hitherto unrecognized and dynamic process within the natural history of neoplastic pancreatic disease, one that apparently consolidates the conflicting evidence observed in previous studies: while there is progressive loss of epithelial primary cilia in ducts from normal pancreas to PanIN lesions of different grades of atypia and finally to PDAC, it is accompanied by a simultaneous increase of PC within stromal cells. The redistribution of PC from the epithelium to the stroma is also consistent with the observation that downstream HH pathway activity is mainly identified within, or even restricted to, the stromal compartment of pancreatic cancer [37].

Interestingly, these findings confirm a recent observation of Emoto and colleagues who described a group of PDAC (25%) expressing epithelial PC; those patients had a worse prognosis than patients without expression of epithelial PC [38]. Two out of 13 PDAC in our cohort also demonstrated a higher PC expression, although it was not histological distinguishable (morphological, grading) from the other 11 PDAC.

The mechanisms of epithelial PC loss are still unknown. An increased proliferation of tumor epithelia may be one explanation, since it has been previously demonstrated and reiterated here (see **Figs 1C** and **7C**) that during mitosis PC are absorbed and used as the spindle apparatus [39]. Mutation of KRAS was also reported to be associated with PC loss [23]. In a murine tumor cell line without PC-bearing cells, inhibition of the mutated KRAS signaling pathway lead to re-development of PC. Kanda *et al*. found no KRAS mutations in healthy pancreatic ducts but in more than 90% of PanIN lesions (all stages). The number of KRAS mutated epithelial cells increased during PanIN progression [40], which is in concordance with the progressive loss of epithelial PC during PanIN progression in the present study.

The HH signaling pathway receptor PTCH1 was found in normal duct epithelia, but was absent in tumor cells. Although one reason might be the internalization of PTCH receptors in epithelia during activation of HH signaling pathways [41], it is more likely that the negative PTCH staining is due to the loss of epithelial PC. The inhibitory effect of PC on HH signaling pathways [42] might lead to an aberrant activation of HH due to the absence of epithelial PC in PDAC. However, investigations by Yauch *et al*. [43] and Tian *et al*. [37] support the theory that ligand-dependent HH signals may not be mediated by epithelial cells, but rather paracrine HH signaling through secretion of soluble HH from epithelial cells [9] activating the identical signaling pathways in ciliated PSC in the stroma [37, 43]. This is supported by our findings showing PTCH2 and SMO receptors in stromal PC and cells of pancreatic neoplasia.

It has been reported that the length of cilia changes in conditions such as injury [44] or inflammation [45], influences cell cycle time [19, 46] and that shortening of PC induces a phosphorylation signaling cascade [47]. It has been therefore been speculated that the information of axonemal length may be sent into the cytosol and that this signal may be able to regulate the cell cycle re-entry [48], nominating PC as a tumor suppressor that potentially transmits cytostatic signals to the cell [39].

A potentially counter-indicative obstructive study in a rat CP model was performed, in which the growth of PC within the pancreatic ducts was observed [49]. However, this was explained as a high intra-luminal pressure in CP and the authors also described a gain of PC length towards bigger ducts. We found a decrease of PC length towards bigger ducts, but these observations in rat cannot be confirmed in humans by this study so the transferability from rodent models to human may be questionable in this case.

Interestingly, a highly significant gain of PC was also seen, particularly in fibrotic areas of the pancreas, independent of ducts. Normal pancreatic tissue contains small areas of pancreatic stellate cells (PSC) as well as a small number of PC. In CP, where stroma becomes desmoplastic [50], there is a significant increase of PC. PDACs demonstrated the highest percentage of stromal PC. This observation correlates with a known property of pancreatic cancer, which undergoes the strongest desmoplasia in comparison to other tumor entities [51].

PSC (on which primary cilia have been detected) have been linked to the initiation of stromal reaction in PDAC and CP [36], therefore it would seem logical that the increase of PC in PDAC and CP stroma may be triggered through pancreatic stellate cells, since these cells also promote tumor cell proliferation [43, 52]. This is consistent with our remarkable observation of very long and numerous PC in fibrotic areas/tubular complexes of CP. Tubular complexes were shown to be part of acinar regeneration, indicating a proliferating area [9]. It seems possible that the increased surface area, and therefore, number of receptors on PC, may be part of the proliferative activity in CP as well as neoplasia.

Alternatively, the reason for this increase of stromal PC may also be modified blood supply in desmoplastic stroma [51], a phenomenon which is consistent with the results of our simulated tumor environment. This desmoplasia within the stroma affects the efficacy of existing chemotherapy, which is limited [53] due to the strong stromal reaction caused by PSC, impeding the accessibility of tumor cells to cytotoxic drugs [54, 55], and extensive development of stromal reaction has been correlated with an unfavorable prognosis [56]. However, it has been recently demonstrated that the stroma is also able to inhibit tumor growth, e.g. by reducing neo-angiogenesis [57, 58]. As such, the PC of the pancreatic stellate cells of the stroma represents a high-yield area for future research.

In conclusion, this study explains the seeming loss of PC in PDAC instead as redistribution from epithelial to stromal PC during PanIN progression to pancreatic cancer. While evidence of the Hedgehog pathway downstream activity in PDAC indicates that primary cilia mediate the stromal reaction (desmoplasia), the function of PC and exact role in pathogenesis are still to be elucidated.
